# 104 Impact of a School-Based Physical Activity Intervention program on Physical Activity Levels, Sleep Patterns, Aerobic Fitness, and Motor Competence

**DOI:** 10.1093/eurpub/ckae114.078

**Published:** 2024-09-26

**Authors:** Júlio A Costa, Susana Vale, Rita Cordovil, Luís P Rodrigues, Vasco Cardoso, Rui Proença, Manuel Costa, Carlos Neto, João Brito, José Guilherme, André Seabra

**Affiliations:** Portuguese Football Federation, Portugal Football School, Oeiras, Portugal; Politécnico do Porto - Escola Superior de Educação, Porto, Portugal, Porto, Portugal; CIAFEL/ITR - Universidade do Porto, Porto, Portugal, Porto, Portugal; CIPER, Faculdade de Motricidade Humana, Universidade de Lisboa, Lisboa, Portugal, Lisboa, Portugal; Faculdade de Motricidade Humana, Universidade de Lisboa, Lisboa, Portugal, Lisboa, Portugal; Instituto Politécnico de Viana do Castelo, Escola Superior de Desporto e Lazer, SPRINT, Melgaço, Portugal, Melgaço, Portugal; Portuguese Football Federation, Portugal Football School, Oeiras, Portugal; School D. Carlos I, Sintra, Portugal, Sintra, Portugal; Portuguese Football Federation, Portugal Football School, Oeiras, Portugal; Faculdade de Motricidade Humana, Universidade de Lisboa, Lisboa, Portugal, Lisboa, Portugal; Portuguese Football Federation, Portugal Football School, Oeiras, Portugal; Portuguese Football Federation, Portugal Football School, Oeiras, Portugal; Faculty of Sport, Centre of Research, Education, Innovation and Intervention in Sport, University of Porto, Porto, Portugal, Porto, Portugal; Portuguese Football Federation, Portugal Football School, Oeiras, Portugal

## Abstract

**Introduction:**

The “Super Quinas” project evaluated the effectiveness of an intervention program to improve PA, aerobic fitness, sleep, and motor competence on children in primary school.

**Methods:**

This pilot project was conducted in 44 schools across the country (i.e., in Portugal), and involved over 1600 school-age children (6 to 10 years old). All 1600 children completed the MC assessment (MCA) in the PRE test in the 1st week and the POST test in the 12th week. Additionally, a sub-group of 38 children underwent more specific tests (i.e., PA, sleep, body composition and aerobic fitness) for a detailed analysis of the impact of this intervention project. The experimental group (n = 19) enrolled in a 12-week intervention program (one more extra-curricular activity class of 60 min per week) compared to the CG (n = 19), all aged 9–10 years. PA and sleep were measured by accelerometry, and aerobic fitness was measured by Children’s Yo-Yo test (YYIR1C) during the 1st week (PRE), the 6th week (DUR), and the 12th week (POST) of the intervention program. MC in PRE and POST intervention was also assessed by the MC Assessment (MCA) instrument. Heart rate (HR, assessed using HR monitors), and enjoyment level were recorded during all intervention program classes.

**Results:**

Comparing the EG and CG in DUR and POST, the EG spent ∼18min and ∼34min more time in moderate to vigorous PA (MVPA) per day (p < 0.001); had ∼44min and ∼203min less sedentary time per day (p < 0.001); performed more 44 and 128m in YYIR1C compared to CG (p < 0.001) and slept more 17 and 114min per night (p < 0.001). In POST MC was significantly better (27%) in the EG compared to CG (p < 0.001). The %HRmax during the extra-curricular classes ranged between 65 and 81% (i.e., light to moderate intensities), and the enjoyment between fun and great fun.

**Conclusion:**

Our findings suggest that adding one more extra-curricular activity class of 60 min per week for 12 weeks effectively increased the levels of PA, aerobic fitness, sleep duration, and MC in children aged 9–10 years.

